# Safety and efficacy of sirolimus in recurrent intravenous leiomyomatosis, pulmonary benign metastatic leiomyomatosis, and leiomyomatosis peritonealis disseminata: a pilot study

**DOI:** 10.1186/s12916-024-03344-9

**Published:** 2024-03-13

**Authors:** Guorui Zhang, Rong Fan, Hua Yang, Hao Su, Xin Yu, Yutong Wang, Fengzhi Feng, Lan Zhu

**Affiliations:** Department of Obstetrics and Gynecology, National Clinical Research Center for Obstetric & Gynecologic Diseases, Peking Union Medical College Hospital, Chinese Academy of Medical Sciences and Peking Union Medical College, Fengzhi Feng, No. 1, Shuaifuyuan, Beijing, 100730 Wangfujing China

**Keywords:** Sirolimus, mTOR inhibitor, Intravenous leiomyomatosis, Pulmonary benign metastatic leiomyomatosis, Leiomyomatosis peritonealis disseminata

## Abstract

**Background:**

Intravenous leiomyomatosis (IVL), pulmonary benign metastatic leiomyomatosis (PBML), and leiomyomatosis peritonealis disseminata (LPD) are leiomyomas with special growth patterns and high postoperative recurrence rates. We report the safety and efficacy of a pilot study of sirolimus in the treatment of recurrent IVL, PBML, and recurrent LPD.

**Methods:**

This was a pilot study to evaluate the safety and efficacy of sirolimus in the treatment of leiomyomatosis (ClinicalTrials.gov identifier NCT03500367) conducted in China. Patients received oral sirolimus 2 mg once a day for a maximum of 60 months or until disease progression, intolerable toxicity, withdrawal of consent, or investigator decision to stop. The primary end point of this study was the objective response rate. Secondary end points included safety and tolerability, disease control rate, and progression-free survival.

**Results:**

A total of 15 patients with leiomyomatosis were included in the study, including five with recurrent IVL, eight with PBML and two with recurrent LPD. The median follow-up time was 15 months (range 6–54 months), nine patients (60%) had treatment-related adverse events (including all levels), and two patients had treatment-related grade 3 or 4 adverse events. The objective response rate was 20.0% (95% CI, 7.1–45.2%), and the disease control rate was 86.7% (95% CI, 62.1–96.3%). Partial response was achieved in three patients. The median response time in the three partial response patients was 33 months (range 29–36 months), and the sustained remission time of these three patients reached 0, 18, and 25 months, respectively.

**Conclusions:**

Sirolimus was safe and effective in the treatment of recurrent IVL, PBML, and recurrent LPD.

**Trial registration:**

ClinicalTrials.gov identifier NCT03500367. Registered on 18 April 2018.

## Background

Intravenous leiomyomatosis (IVL), pulmonary benign metastatic leiomyomatosis (PBML), and leiomyomatosis peritonealis disseminate (LPD) are rare leiomyomas with special growth patterns. Histologically, they present with a benign leiomyoma appearance with unique growth sites and patterns. IVL usually originates from the uterus, spreads along the blood vessels to the proximal end, and occasionally affects the heart. Its clinical manifestations mainly include symptoms similar to uterine myoma and symptoms related to decreased right heart function [1, 2]. Depending on the range of the lesions, surgeries might be achieved through multidisciplinary collaboration, including gynecologists, vascular surgeons, and cardiac surgeons, with the aim of complete resection [3]. However, the postoperative recurrence rate is as high as 14–31.0% [1, 4, 5]. Benign metastatic leiomyomatosis refers to the presence of benign leiomyomas outside the uterus, which often occurs in patients who have previously undergone myomectomy or hysterectomy. The lung is the most commonly affected site, but the rarely affected sites include bone, spine, lymph nodes, retroperitoneum, and heart [6]. LPD is characterized by multiple nodules on the peritoneum, without special symptoms [7]. It is usually found incidentally during laparoscopy, transabdominal surgery, or cesarean section [8]. The nonspecific symptoms mainly include abdominal pain, vaginal bleeding, abdominal mass, and gastrointestinal discomfort. The pathogenesis of IVL, PBML, or LPD is unclear. Histologically, they present as benign smooth muscle. The number of mitotic figures per high-power field is less than 5, without nuclear atypia or necrosis, suggesting that the tumor is benign.

Surgery is the main treatment for IVL and LPD [9]. PBML progresses slowly. Surgery is unnecessary for patients without symptoms as long as they are regularly followed up [10], while patients with symptoms are suggested to receive surgical resection. It is generally believed that IVL, PBML, and LPD usually express estrogen receptors or progesterone receptors. Therefore, bilateral oophorectomy and/or antiestrogen therapy are the most common adjuvant treatment options, especially for patients who are not suited for surgery removal or are unwilling to accept surgery. However, the effect of antiestrogen therapy has not been satisfactory [1], and there is insufficient evidence to support the antiestrogen therapy in patients with incomplete resections or recurrence [11]. The postoperative recurrence rate is high. In addition, the adverse effects of antiestrogen therapy are severe. It can cause vasoconstriction symptoms such as hot flashes and sweating in the short term and may lead to problems such as osteoporosis in the long term.

Mammalian target of rapamycin (mTOR) inhibitors may become an effective treatment option for leiomyomatosis. mTOR, a master regulator of proliferation, is activated in many tumors, possibly by mechanisms that are similar to some human fibrosis syndromes and/or by mutation of upstream tumor suppressor genes [12]. Transcriptome analysis has found that the target of rapamycin (mTOR) pathway was the most significantly upregulated pathway in uterine leiomyoma [13]. Inhibition of mTOR with the rapamycin analogue WAY-129327 significantly decreased tumor incidence, multiplicity, and size [14]. Here, we report the safety and efficacy of sirolimus (mTOR inhibitor) in the treatment of recurrent IVL, PBML, and recurrent LPD.

## Methods

### Study design and participants

This study (ClinicalTrials.gov identifier NCT03500367) was a pilot study to evaluate the safety and efficacy of sirolimus in the treatment of leiomyomatosis, which was conducted in China between 2018 and 2022. The study protocol was approved by the Institutional Review Board of Peking Union Medical College Hospital, and written informed consent was obtained from each participating patient.

The main inclusion criteria were (1) pathological or imaging diagnosis of leiomyomatosis with special growth patterns, including IVL, PBML, and LPD; (2) not suited or unwilling to receive surgery; (3) hoping for pharmaceutical treatment; (4) age over 18 years; (5) measurable disease at baseline under the Response Evaluation Criteria in Solid Tumor (RECIST) version 1.1; (6) an Eastern Cooperative Oncology Group performance status of 0 or 1; and (7) adequate organ function within 10 days of treatment initiation. The main exclusion criteria were desire for pregnancy, malignant tumor, severe anemia, and severe coagulation dysfunction. Withdrawal criteria included sirolimus drug allergy, serious adverse events, and patient’s wish to withdraw.

### Treatment and evaluation

Patients received oral sirolimus capsule (Yixinke®, North China Pharmaceutical Group Corporation) 2 mg once daily for a maximum of 60 months or until disease progression, intolerable toxicity, withdrawal of consent, or investigator decision to stop it. The drug concentration was monitored by measuring the concentration of sirolimus in peripheral venous blood at 1 month, 3 months, 6 months, 9 months, and 12 months for the first year and then every 6 months until 5 years after sirolimus administration. Dosage was adjusted to maintain trough concentration between 5 and 15 ng/ml. If a patient had severe adverse reactions and failed to tolerate the drug, her dosage could be reduced to a minimum dose of 1 mg per day. Safety was assessed throughout the study and for 90 days after treatment discontinuation. Adverse events were graded using the National Cancer Institute Common Terminology Criteria for Adverse Events (version 4.0). For patients exhibiting grade 4 treatment-related toxicities, treatment could be discontinued. Tumor response was assessed using computed tomography or magnetic resonance imaging every 3 to 6 months for the first year and every 6 to 12 months thereafter. If imaging indicated progressive disease, a confirmatory assessment was required 4 weeks later. Patients could continue receiving the study treatment during this period. If the repeat scan confirmed progression, the study treatment was discontinued.

### End point

The primary end point of this study was the objective response rate (ORR) as determined by investigator review, defined as the proportion of patients with a best overall response of confirmed complete response or partial response per RECIST. Secondary end points were safety, tolerability, disease control rate (DCR; defined as complete response, partial response or stable disease per RECIST), and progression-free survival (PFS; defined as time from allocation to the first documented disease progression according to RECIST) [15]. Efficacy was assessed per RECIST in all patients who took oral sirolimus for over 3 months. Safety was assessed in all patients who took oral sirolimus for over 1 month.

### Statistical analysis

ORR, PFS, and DCR were analyzed using the Kaplan–Meier method, and safety data were summarized using descriptive statistics. Statistical analyses were performed by SPSS 18.0. The data cutoff for this analysis was December 31, 2022.

### Data availability

The data generated in this study are available upon request from the corresponding author.

## Results

### Participants

**Fig. 1:**
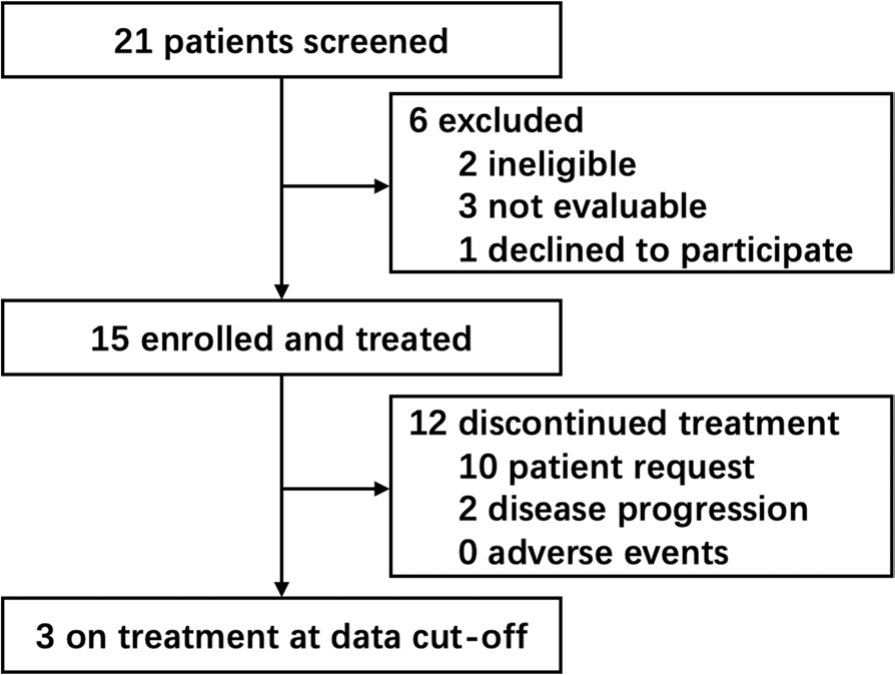
Trial profile

From January 1, 2018, to June 30, 2022, a total of fifteen patients with leiomyomatosis were enrolled in the study (Fig. 1), including five cases of recurrent IVL, eight cases of PBML, and two cases of recurrent LPD. All fifteen patients received sirolimus treatment. The cutoff date for the analysis was December 31, 2022. The median duration of follow-up was 15 months (range 6–54 months). By the cutoff, three patients were still on treatment. Treatment was discontinued due to patient request (*n* = 10), disease progression (*n* = 2), and adverse events (*n* = 0). One of the enrolled patients was previously reported in a BJOG case report [16], but in this study, the patient’s medication and follow-up time were extended to 54 months.

The baseline data of all patients are shown in Table 1. The median age was 46 years. Eleven patients had previously received total hysterectomy, and nine patients had received bilateral oophorectomy. Of the five recurrent IVL patients, all had received hysterectomy and bilateral oophorectomy, and their IVL lesions presented as a recurrent pelvic mass. Of the eight PBML patients, six had previously received hysterectomy and four had received bilateral oophorectomy. Of the two patients with recurrent LPD, one had previously received hysterectomy, and the ovaries were preserved in both cases. The five recurrent IVL patients and the two patients with recurrent LPD were diagnosed pathologically after the primary surgery. Four PBML cases were diagnosed pathologically by needle biopsy of lung masses, and four PBML cases who failed to biopsy were diagnosed by imaging and previous history of uterine leiomyoma. Two patients received sirolimus treatment and letrozole treatment simultaneously.

**Table 1:** Baseline patients characteristics (*N* = 15)

Characteristic	Patients (%)
Age, years, median (range)	46 (34–59)
ECOG performance status score	
0	12 (80.0)
1	3 (20.0)
Disease	
Intravenous leiomyomatosis	5 (33.3)
Pulmonary benign metastatic leiomyomatosis	8 (53.3)
Leiomyomatosis peritonealis disseminata	2 (13.3)
Previous hysterectomy	
Yes	11 (73.3)
No	4 (26.7)
Previous bilateral oophorectomy	
Yes	9 (60.0)
No	6 (40.0)

*Abbreviation*: *ECOG*, Eastern Cooperative Oncology Group

### Antitumor activity

**Fig. 2:**
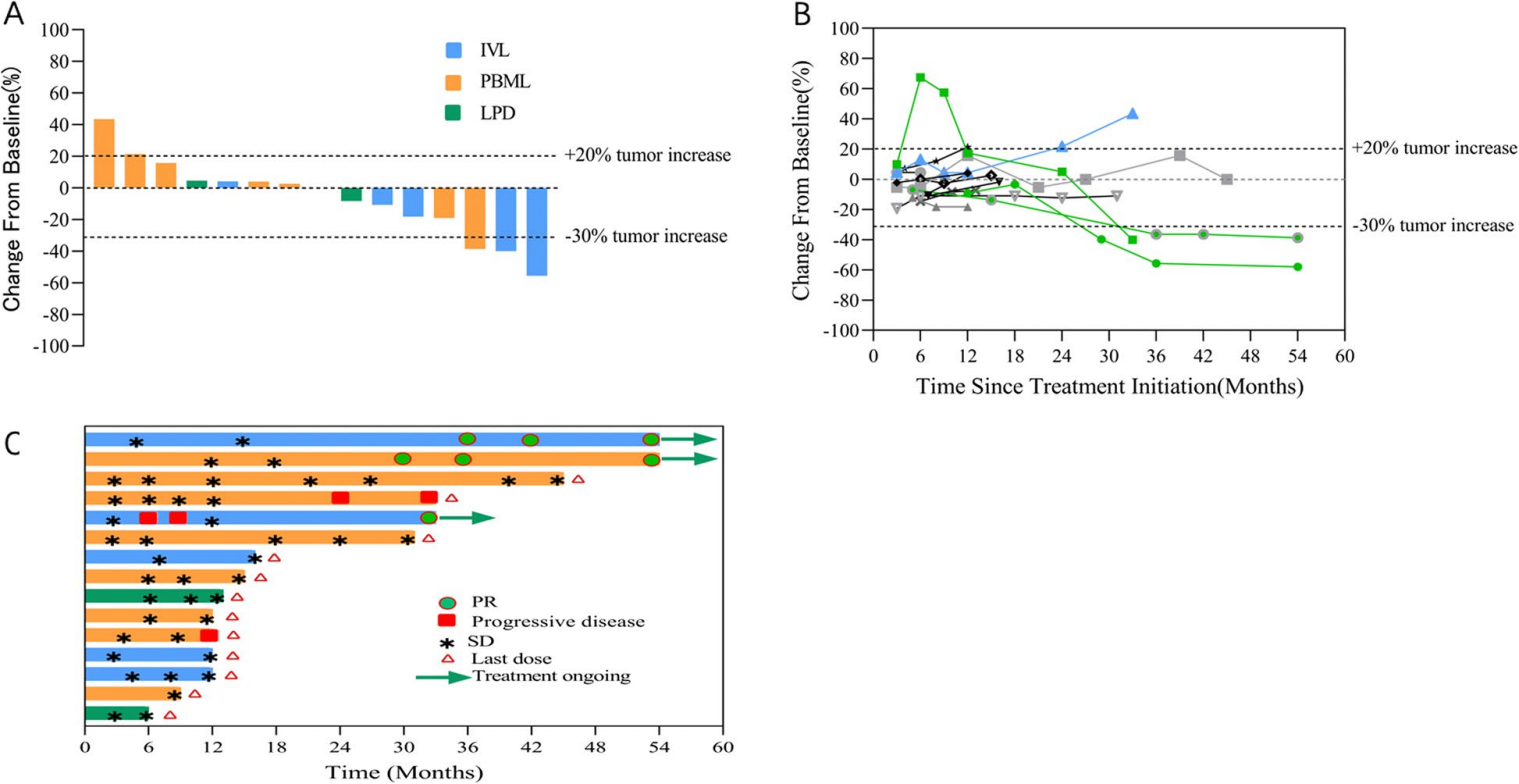
Antitumor activity of sirolimus. **A** Changes in target lesions at the time of best response (*n* = 15). **B** Longitudinal changes in tumor size from baseline (*n* = 15). **C** Treatment exposure and response duration (*n* = 15)

Fifteen patients were included in the efficacy analysis. The ORR following RECIST (version 1.1) as determined by investigator review was 20.0% (95% confidence interval (CI), 7.1–45.2%), and the DCR was 86.7% (95% CI, 62.1–92.3%) (Table 2 and Fig. 2). Partial response was achieved in three patients, including two recurrent IVL patients and one PBML patient. Stable disease was recorded in ten cases, including the two patients receiving sirolimus and letrozole simultaneously. Progressive disease was recorded in two cases, both PBML.

**Table 2:** Best objective response assessed per RECIST (version 1.1) by investigator review (*n* = 15)

Best objective response	No	%	95% CI
ORR	3	20.0	7.1–45.2
DCR	13	86.7	62.1–96.3
PR	3	20.0	7.1–45.2
SD	10	66.6	41.7–84.8
PD	2	13.3	3.7–37.9

Compared with baseline, the tumor size decreased in seven cases, and this decrease was durable in the patients demonstrating partial response. The median response time of the three partial response patients was 33 months (range 29–36 months), and the sustained remission time of these three patients reached 0, 18, and 25 months, respectively (still in continuous treatment).

The median PFS of all fifteen patients was not reached, and the PFS rates at 12 months and 24 months were 92.3% and 76.9%, respectively.

### Safety in this case series

All fifteen patients were included in the safety analysis. Nine patients (60%) had treatment-related adverse events (including all levels), as shown in Table 3. Adverse events with an incidence rate of more than 10% included oral ulcers (*n* = 5, 33.3%) and liver enzyme elevation (*n* = 2, 13.3%). Two patients had treatment-related adverse events ≥ grade 3 (one case of severe oral ulcer and one case of severe maculopapule). There were no grade 4 adverse events. Among all patients, concentrations of sirolimus were higher than 15 ng/ml in two patients at 9 months and 12 months after sirolimus administration, separately, and grade 3 adverse events were observed in the two patients. Thus, the sirolimus dose for the two patients was reduced to 1 mg once daily. The sirolimus concentrations of the other 13 patients were all within the normal range. Drug administration in one case was delayed for two months due to adverse events. No treatment-related deaths occurred.

**Table 3:** Treatment-related adverse events

Adverse events	Any grade No.(%)	Grade 1/2 No.(%)	Grade 3/4 No.(%)
Any	9 (60.0)	7	2
Mouth ulcer	5 (33.3)	4	1
Elevated liver enzymes	2 (13.3)	2	0
Dyslipidemia	1 (6.67)	1	0
Maculopapule	1 (6.67)	0	1

### Exploratory analysis

**Fig. 3:**
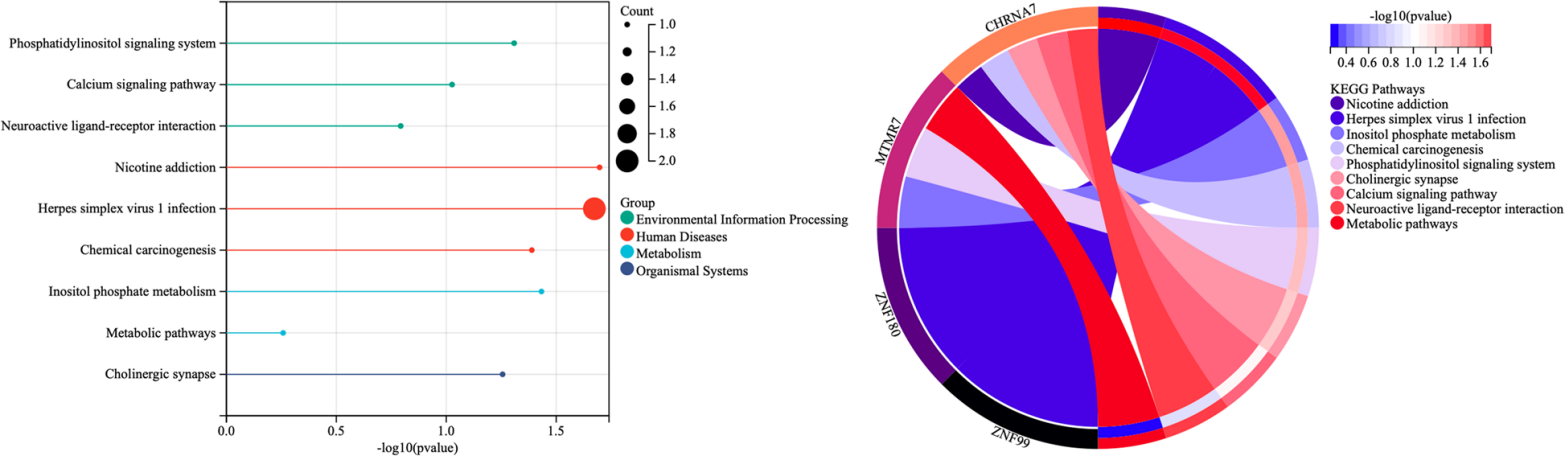
Bubble plot (**A**) and circle plot (**B**) of the top 9 KEGG pathways

For the exploratory analysis of the targets and mechanisms of sirolimus, five tumor tissue samples from four patients were obtained for gene sequencing (pelvic and intracardiac tumor samples were tested separately in an IVL patient). Whole-exon sequencing was performed on four patients, including one PR patient (an IVL patient) and three SD patients (two IVL patients and one PBML patient). In the whole-exon sequencing of the PR patient, a total of 20 mutated genes were found to coexist in both pelvic and intracardiac tumor samples. Signaling pathway enrichment analysis based on KEGG was performed with the 20 mutated genes to identify the common functions and related pathways of the mutated gene set. The nine enriched KEGG signaling pathways were displayed in bubble and circle plots (Fig. 3). The results indicated that the phosphatidylinositol signaling system might be related to the mechanism of sirolimus in the patient. Estrogen-dependent signaling pathways can be classified as genomic and nongenomic. Nongenomic pathways are typically mediated through rapid activation of signaling cascades. The phosphatidylinositol signaling system is an important pathway of estrogen action in the pathogenesis of uterine fibroids.

## Discussion

In this study, sirolimus was administered for recurrent IVL, PBML, and recurrent LPD. In the safety evaluation, two patients had treatment-related adverse events ≥ grade 3. In the efficacy evaluation, the ORR was 20.0%, and the DCR was 86.7%. The results suggest that sirolimus is a promising treatment alternative for recurrent IVL, PBML, and recurrent LPD.

Uterine fibroids are benign tumors derived from uterine smooth muscle cells. Its pathological mechanism is still unclear. Genetic susceptibility and exposure to steroid hormones and growth factors might promote the formation and growth of fibroids [17]. Some rare types of uterine fibroids, including IVL, PBML, and LPD, are difficult to treat because of the wide spread of the tumors, and sometimes a single operation cannot achieve radical resection [9]. Generally, uterine fibroids are estrogen-dependent tumors. Medication treatment, including gonadotropin-releasing hormone analogues, progesterone receptor antagonists, and combined oral contraceptives, is mainly used to shrink fibroids, inhibit the growth of fibroids, and prevent disease deterioration [18]. However, their efficacy varies greatly, and recurrence is common after drug withdrawal, resulting in short-term use in most situations.

mTOR inhibitors have therapeutic effects against cancer development and progression [19]. mTOR is a conserved serine-threonine kinase that regulates protein synthesis in transcription and translation through a variety of target molecules, promoting cell growth and proliferation [12]. mTOR inhibitors showed antitumor responses in benign tumors of the tuberous sclerosis complex (TSC), including lymphangiomyomatosis, renal angiomyolipoma, cardiac rhabdomyoma, facial angiofibroma, and retinal astrocytic hamartoma [20]. TSC is caused by mutation of the tumor suppressor genes (TSC1 or TSC2 with protein products of hamartin and tuberin, respectively) heterodimerizing and negatively regulating the activation of mTOR. An increasing number of studies have reported the exceptional effectiveness of sirolimus in treating some rare tumors, including kaposiform hemangioendothelioma [21].

Currently, there was no research or report on the effect and possible mechanism of sirolimus in leiomyomatosis in the literature. Researchers had made some attempts in exploring the pathogenesis of IVL, although no consensus has been formed. Recurrent chromosome alterations detected in IVL overlap with those observed in the spectrum of uterine fibroids and involve genes implicated in mesenchymal tumors, with the most common genetic alterations involved 1p (39%), 22q (36%), 2q (29%), 1q (25%), 13q (21%), and 14q (21%) [22]. The exploratory analysis of this study suggested that sirolimus might exert its effects by regulating the phosphatidylinositol signaling system, a signal pathway relating to the estrogen action. Therefore, we comprehensively reviewed the researches relating to the effect of sirolimus on uterine fibroids and boldly speculated on the potential mechanism of sirolimus on leiomyomatosis. Of course, further researches were needed to validate the following possible actions on leiomyomatosis.

The mTOR inhibitor sirolimus might become an effective treatment option for uterine fibroids and special types of leiomyomatosis. An unbiased pathway analysis using a method of gene-set enrichment based on the sigPathway algorithm detected the mTOR pathway as one of the most highly upregulated pathways in uterine leiomyoma cells [13]. Inhibition of mTOR with the rapamycin analogue WAY-129327 significantly decreased tumor incidence, multiplicity, and size [14]. Rapamycin-induced growth arrest required a sense barrier that could be impaired by telomerase [23]. Animal experiments confirmed that the mTOR inhibitor rapamycin (sirolimus) significantly reduced the growth rate of uterine leiomyoma transplanted subcutaneously in rats [24]. Rapamycin inhibited myoma smooth muscle cell growth at a low concentration (0.01 mg/mL). However, there is currently no clinical research on mTOR inhibitors in the treatment of uterine fibroids in humans.

Whole-exon sequencing in this study showed that the therapeutic effect of sirolimus on leiomyomatosis was achieved by regulating the phosphatidylinositol signaling system. Uterine fibroids are estrogen-related tumors [25]. In vitro experiments have confirmed that estradiol signaling can integrate a variety of extracellular signals and regulate various cellular functions, including cell growth, differentiation, transformation, and survival. Estradiol binds to the estrogen receptor and activates protein kinase pathways such as mitogen-activated protein kinase (MAPK) through the Ras-Raf-MEK-MAPK pathway and phosphatidylinositide 3-kinases (PI3K)–Akt through the PI3K–phosphatidylinositol-3,4,5-trisphosphate (PIP3)–Akt–mTOR pathway [25–27]. In the PI3K-PIP3-Akt-mTOR pathway, the binding of estradiol to receptors leads to the activation of PI3K, which in turn phosphorylates the plasma membrane lipid phosphatidylinositol-4,5-diphosphate to PIP3. This process leads to the recruitment and activation of Akt proteins, which regulate mTOR, glycogen synthase kinase 3, and other proteins [27]. This pathway regulates important processes, including the cell cycle, proliferation, and survival. Abnormal PI3K-PIP3-Akt-mTOR pathway activation might be related to the pathogenesis of fibroids. Scholars have described the upregulation of mTOR signaling in fibroids in humans and animal models [13]. Another group found that the expression of glycogen synthesis kinase-3 and cyclin D2 was higher in fibroids than in the uterine myometrium [28]. Sirolimus (an mTOR inhibitor) might exert tumor inhibitory effects on leiomyomatosis through the PI3K–PIP3–Akt–mTOR signaling pathway.

This study was the first prospective pilot study to explore the efficacy and safety of sirolimus in the treatment of recurrent IVL, PBML, and LPD. The results showed that some patients achieved PR. However, this study has several limitations. Firstly, recurrent IVL, PBML, and LPD are rare in clinical practice, making it difficult to recruit a large sample. After 3.5 years, only fifteen patients were included. Secondly, during patient enrollment, we recommended that patients should receive imaging assessment every 3 months within the first year of medication, but in practice, we found that it was hard to achieve. IVL, PBML, and LPD usually progressed slowly, and some patients went through a period of observation before enrolling in this study. Thus, some patients believed that the lesions would not show significant changes after 3 months and were not willing to receive imaging examinations every 3 months. In addition, IVL, PBML, and LPD were rare in clinical practice. The long distances between the patient’s place of residence and our medical center in some patients posed difficulties for frequent postoperative follow-up. Therefore, our final implementation guidelines suggested that patients received imaging assessment every 3 months within the first year of medication, with a maximum acceptable interval of every 6 months. The results showed that 7 patients received imaging assessments every 3 months, and 8 patients received it every 6 months, within the first year of medication. Thirdly, the scope and depth of genetic sequencing were insufficient. Only four of the fifteen enrolled patients underwent genetic sequencing. The small amount of biopsy tissue taken from PBML patients and the long storage time of tissue samples from some recurrent IVL patients affected the accuracy of gene sequencing. Finally, the cohort enrolled different subtypes of leiomyomas, with variable clinical characteristics, possibly increasing bias. The results of this study showed that sirolimus might have a better effect in IVL patients. Multicenter, prospective IVL cohort studies with larger samples are warranted. In addition, to explore the mechanism and target of sirolimus in IVL, peripheral blood cell-free DNA sequencing might provide new insight.

## Conclusions

Sirolimus is safe and effective in the treatment of recurrent IVL, PBML, and recurrent LPD. Its clinical application value needs to be confirmed by larger clinical studies.

## Data Availability

All data requests should be submitted to the corresponding author for consideration.

## Abbreviations

IVL: Intravenous leiomyomatosis
PBML: Pulmonary benign metastatic leiomyomatosis
LPD: Leiomyomatosis peritonealis disseminate
mTOR: Mammalian target of rapamycin
RECIST: Response Evaluation Criteria in Solid Tumor
ORR: Objective response rate
DCR: Disease control rate
PFS: Progression-free survival
MAPK: Mitogen-activated protein kinase
PI3K: Phosphatidylinositide 3-kinases
PIP3: Phosphatidylinositol-3,4,5- trisphosphate
MED12: Mediator complex subunit 12
